# Clinical Characteristics and Outcomes of Interstitial Lung Disease in Primary Sjögren's Syndrome: A Retrospective Cohort Study

**DOI:** 10.31138/mjr.230323.cca

**Published:** 2024-01-16

**Authors:** Prathyusha Manikuppam, Shivraj Padiyar, Bijesh Yadav, Avinash A. Nair, Manisha Mane, John Mathew

**Affiliations:** 1 Department of Clinical Immunology and Rheumatology, Christian Medical College, Vellore, India,; 2 Department of Biostatistics, Christian Medical College, Vellore, India,; 3 Department of Pulmonary Medicine, Christian Medical College, Vellore, India,; 4 Department of Radiology, Christian Medical College, Vellore, India

**Keywords:** interstitial lung disease, mycophenolate mofetil, spirometry, Sjögren's syndrome

## Abstract

**Objectives:**

To describe the characteristics of primary Sjögren's syndrome (pSS) patients with interstitial lung disease (ILD) and to assess treatment response.

**Methods:**

All patients of pSS from 2010 to 2019 were retrospectively identified. Lung function tests, high resolution computed tomography (HRCT) findings, and treatment outcomes were analysed.

**Results:**

Out of 550 patients with pSS, ILD was detected in 33 patients (frequency of 6 %). The mean(±SD) age at the diagnosis of pSS was 50 (± 9.3) years. 28/33(84.8%) were females. ILD onset preceded pSS diagnosis in 2 (6%) patients, simultaneously diagnosed in 21 (63.6%) patients and developed after pSS onset in 10 (30.3%) patients. 5 patients (15.15 %) were asymptomatic for ILD. Non-specific interstitial pneumonia (NSIP) accounted for the most frequent ILD subtype, in 15 patients (45.5%). Mycophenolate mofetil (MMF) was the most frequently used steroid sparing agent, in 25 patients (75.7%). 7 patients were lost to follow up. Response was seen in 22 patients, whereas 3 patients were non responders. There was one mortality due to lower respiratory tract infection-related sepsis. Presence of sicca symptoms [91.5% vs 8.7% (p<0.001)], NSIP pattern of ILD [90% vs 10% (p = 0.002)], and absence of Raynaud's phenomenon [91.7% vs 8.3% (p<0.001)] were significantly associated with responder status when compared to non-responders.

**Conclusion:**

ILD in primary Sjögren's syndrome is not an uncommon entity, and immunosuppression with steroids along with steroid-sparing agents led to good clinical outcomes of ILD in a majority of the patients in our cohort.

## INTRODUCTION

Primary Sjögren's syndrome (pSS) is a systemic autoimmune disorder, characterised by sicca symptoms, a wide spectrum of organ involvement, and lymphocytic infiltration of exocrine glands on histopathology.^1^ The disease is common in middle-aged women, with a varied prevalence of 0.1 to 4.8 % and a female-to-male ratio of 9:1.^2^ Extra-glandular involvement can be the presenting manifestation in 5–20% of patients^3^ and can range from arthralgia or arthritis, Raynaud's phenomenon, and fatigue, to the involvement of major organs like lungs, heart, liver, kidneys, bladder, nervous system, or lymphoreticular system.^4^ The prevalence of pulmonary involvement ranges from 9–75% based on the detection modality, and comprises of small airway, parenchymal, and interstitial involvement.^5^ Subclinical involvement is not uncommon.^6^ Pulmonary manifestations include follicular bronchiolitis, bronchiectasis, bronchial hyper-reactivity, interstitial lung disease (ILD), pulmonary amyloidosis, pulmonary lymphoma, pulmonary embolism, pulmonary hypertension, and rarely pulmonary vasculitis, alveolar haemorrhage, and diaphragmatic dysfunction.^6^ The prevalence of ILD varies from 1.5% to 78% according to different studies from different regions.^7^ The association of ILD with pSS was first described in 1973 with lymphocytic interstitial pneumonia (LIP).^8^ There is a strong association between LIP and pSS which can be a precursor to bronchus-associated lymphoid tissue (BALT) lymphoma.^9^ The most common subtype of ILD is nonspecific interstitial pneumonia (NSIP).^10^ The other types of ILD documented were usual interstitial pneumonia (UIP) and organising pneumonia (OP).^10^

Few studies have looked at the risk factors for the development of ILD in pSS and found that there was an increased risk with aging, cigarette smoking, ANA positivity,^11^ rheumatoid factor, C-reactive protein (CRP),^12^ and anti-ro-52 antibodies.^13^ There is limited data on the management of ILD in pSS, with no controlled trials. For symptomatic patients, treatment is initiated with oral corticosteroids. Steroid sparing agents including Azathioprine, Mycophenolate mofetil, and Rituximab have shown improvement in several case series and case reports.^6^,^10^ We hereby present a descriptive analysis of interstitial lung disease in pSS from our centre and the management of the same.

## AIMS AND OBJECTIVES

To describe the clinical, laboratory, and radiological characteristics of primary Sjögren's patients with ILD and to assess treatment response to steroid-sparing agents.

## METHODS

A computer-aided search was done from electronic medical records, to identify patients of pSS with ILD in our tertiary care teaching hospital from January 2010 to August 2019.

**Figure 1:**
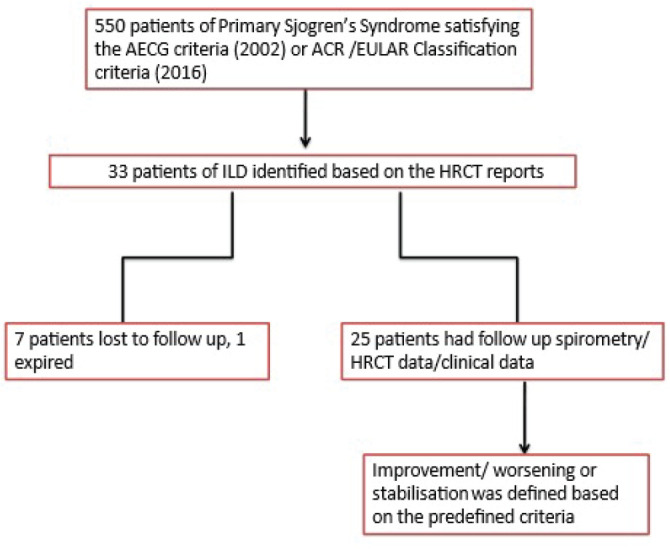
Flowchart of patients recruited for the study.

Patients had to satisfy the 2002 American-European Consensus Group (AECG) criteria for pSS or the 2016 American College of Rheumatology (ACR) / European Alliance of Associations for Rheumatology (EULAR) classification criteria to be included into the study. The flowchart of patients recruited into study is summarised in **Figure 1**.

**Figure 2:**
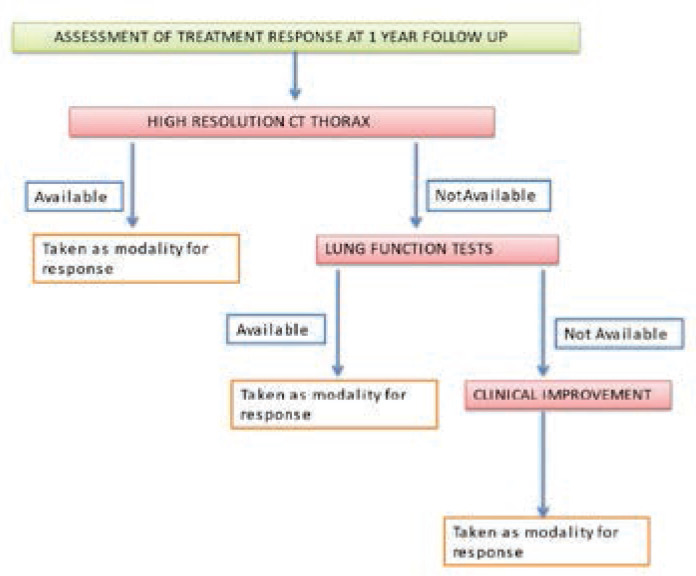
Predefined criteria for improvement.

Demographic data, clinical features, laboratory results, radiologic findings, pulmonary function tests, and the treatment regimens were noted down from the electronic medical records. Patients in our institution underwent high-resolution computed tomography (HRCT) if there were symptoms or signs suggestive of ILD. The diagnosis of ILD required the presence of bilateral parenchymal involvement consistent with ILD. Patients with focal abnormality were excluded. Clinical, radiological, and functional response to treatment at 1 year of follow up was noted. Since all patients did not have spirometry and HRCT at baseline and follow up, a pre-defined protocol for response was devised (**Figure 2**). Radiological response was assessed by a single radiologist who was unaware of the clinical data. Radiological response was categorised into one of the three groups - stable / improved / worsened. In spirometry, assessment of response was done according to the 2019 update on pulmonary function testing by American Thoracic Society.^14^ Improvement was defined as increase in Forced Vital Capacity (FVC) of more than or equal to 10% from baseline, stable disease as less than 10% change in FVC from baseline and worsening as decline in FVC by more than 10% from baseline. In patients with no follow up imaging or spirometry, assessment was done by overall clinical response noted down by the physician at the time of outpatient consultation. The pulmonary domain score of EULAR Sjögren's syndrome disease activity index (ESSDAI) was noted at baseline and follow up.

We have excluded the lost to follow up patients in the outcome analysis to avoid the information bias. As highlighted above, the radiologist was blinded to the treatment and clinical outcomes to avoid detection bias. This study has been approved by the Institutional Review Board of our institution on 29^th^ September of 2021, with waiver of consent obtained due to its retrospective nature (IRB Min. No: 12488).

### Statistical analysis

Descriptive statistics were reported using mean ± standard deviation (SD) or median (inter-quartile range (IQR) as appropriate. A comparison of means was done using two independent-sample t-tests. Association between the categorical variables (CRP, sicca, Raynaud's, type of ILD, duration of ILD) and outcome was established using chi-square and for the continuous variables t-test was used. P<0.05 was considered to be statistically significant. Data were analysed using the Statistical Package for the Social Sciences (SPSS) version 25.

### Results

A total of 550 patients of pSS fulfilling the classification criteria were identified, out of which 33 patients had ILD. The frequency of ILD in pSS in our cohort was 6%. The mean± SD age at the diagnosis of pSS was 50± 9.3 years. 28/33(84.8%) were females. The clinical features are summarised in **Table 1**. ILD onset preceded pSS diagnosis in 2 patients (6%), was concurrently identified in association with pSS in 21 (63.6%) patients, and developed after pSS onset in 10 (30.3%) patients. Sicca symptoms were absent in 4 patients. 25 patients (75.7 %) had positive anti-nuclear antibody (ANA). 28/33 (84.8%) had anti–SSA and 14/26 (53.8%) had anti-SSB antibody positivity. Rheumatoid factor was positive in 12/27 (44.4%) patients. Lip biopsies were performed in 28 patients, 20 (71.4%) of whom had focus score more than or equal to 1. At presentation, 5 patients (15.15 %) were asymptomatic for ILD. NSIP pattern contributed to most cases of ILD, seen in 15 patients (45.5%). UIP and LIP pattern of ILD were seen in 7 (21.2%) and 5 (15.15%) patients, respectively. Organising pneumonia was seen in 1 patient, whereas an unclassifiable pattern was seen in 5 patients. Pulmonary function tests could be retrieved in 19 patients (57.5%) out of whom 13 patients showed restrictive lung defects. Two-dimensional echocardiography was done in 16 patients to look for pulmonary hypertension, of which only 1 patient had echo features suggestive of same.

**Table 1.** Baseline clinical features of patients with pSS.

**Parameters (n=33)**	**No. of patients n (%)**

Age at onset of sicca, years (mean±SD)	50 ± 9.3
	5/28 (84.8%)
Sex (M/F)	
Clinical features	
Parotidomegaly	2 (6%)
Sicca symptoms	29 (87.8%)
Arthritis/arthralgia Raynaud's	14 (42.4%)
Vasculitis	4 (12.12 %)
Baseline ^*^ESSDAI, mean±SD	3 (9.09%)
Pulmonary symptoms	9.39± 2.76
Cough	16 (48.4%)
Dyspnoea	24 (72.7%)
Asymptomatic	5 (15.15%)
Type of ILD	
NSIP	15 (45.5%).
UIP	7 (21.2%)
LIP	5 (15.15%)
OP	1 (3.03%)
Unclassifiable	5 (15.15%)

NSIP: Non-specific interstitial pneumonia; UIP: Usual interstitial pneumonia; LIP: lymphocytic interstitial pneumonia; OP: Organising pneumonia.

* Pulmonary component, ESSDAI: EULAR Sjögren's syndrome disease activity index.

### Treatment outcomes data

20 patients received 0.5mg/kg of prednisolone equivalent, 8 patients received 1mg/kg, 5 patients received 0.25mg/kg or less. One patient received pulse methylprednisolone therapy for vasculitic neuropathy in addition to high-dose corticosteroids. Mycophenolate mofetil (MMF) was the most common immunosuppressant used in 25 patients (75.7%), followed by Azathioprine in 7 patients (21.2%), Cyclophosphamide in 1, and Rituximab along with MMF in 2 patients.

**Figure 3:**
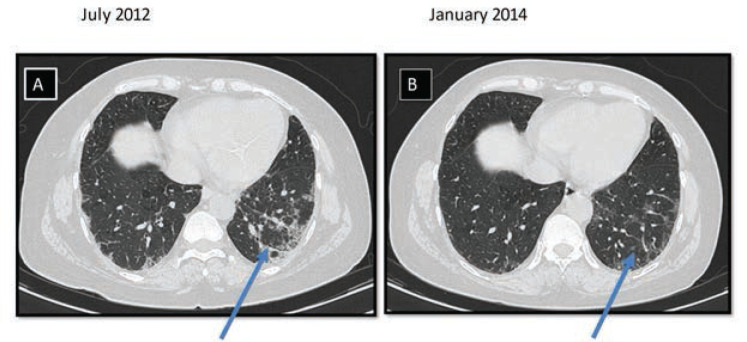
High-resolution CT thorax of a patient at baseline (A) and after 2 years of treatment (B). (A)Shows microcystic honeycombing, bronchiectasis, and traction bronchiectasis at the basal zones of the lung with involvement of subpleural region (B) marked resolution in the lung parenchymal changes after treatment

Patients who had a follow up of at least 1 year were taken for outcome analysis. The mean ± SD duration of follow-up of 33 patients was 41.5 ± 37.8 months. 7 patients were lost to follow-up. There was one mortality due to sepsis because of lower respiratory tract infection, within 1 year of diagnosis of ILD. Criteria based response was seen in 22/25 (88 %) patients and the rest of the three patients were non responders. All 25 patients had baseline HRCT imaging; however, follow-up imaging was available for only 21 patients. At one year of follow up, stable disease in HRCT was seen in 11 (52.4%) patients, regression in 7 (33.3%) patients, and progression of disease was seen in only 3 (14.3%) patients. The HRCT comparison of one of the patients, who responded to therapy is shown in **Figure 3A,B**. The mean ± SD pulmonary domain score of ESSDAI improved from 9.39 ± 2.76 at baseline to 4 ± 3.05 at 1 year of follow up (p<0.001).

Out of 25 patients on MMF, 5 patients were lost to follow up, 1 patient succumbed to lower respiratory tract infection and three patients were non responders requiring additional immunosuppression. Azathioprine was prescribed in 7 patients, of which 2 patients were lost to follow up and 5 patients had criteria-based response. The only one patient on cyclophosphamide met criteria for response. Two patients had received Rituximab, both were given 2 doses of 1 gram, 14 days apart. The indication for the first patient was progression of ILD on MMF, who on follow up attained stable disease at 6 months. This patient had worsening ILD at 1 year follow up, at which point the MMF dose was increased to 3 gram/day. At the last recorded follow-up at the end of 3 years, he was clinically stable, inspite of mild radiological worsening. The second patient received Rituximab for leukocytoclastic vasculitis. The patient had stable ILD on MMF even before the administration of Rituximab. On follow-up with maintenance MMF, ILD remained stable 2 years after Rituximab.

A logistic regression analysis was done to determine the factors predicting treatment response. Presence of sicca symptoms [91.3% (95% CI: 70.5% - 98.5%, p<0.001)], NSIP pattern of ILD [90% (95% CI: 54.1% - 99.5%, p = 0.002)], and absence of Raynaud's phenomenon [91.7% (95% CI: 76% - 99.8%, p<0.001)] were significantly associated with responder status (**Table 2**).

**Table 2.** Logistic regression analysis for predictors of treatment response.

**Variables**	**Group**		**p-value**
	**Non - Responder**	**Responder**	
	**n (%)**	**n (%)**	
Presence of Sicca symptoms	2 (8.7)	21 (91.3)	<0.001
NSIP pattern of ILD	1 (10)	9 (90)	0.002
Absence of Raynaud's	2 (8.3)	22 (91.7)	<0.001
Duration of ILD symptoms, months, mean ± SD	3 ± 2.65	21.48 ± 35.3	0.38
CRP at ILD, mg/L mean ± SD	6.93 ± 6.57	14.64 ± 18.5	0.42

ILD: Interstitial lung disease; NSIP-Non-specific interstitial pneumonia; CRP: C-reactive protein.

## DISCUSSION

In this retrospective study, we had identified 33 patients of primary Sjögren's syndrome with ILD. The 6 percent frequency in our cohort was slightly lower than the previous cohorts reported in the literature which is ranging from 10–20 percent.^15–17^ NSIP pattern was the most common type (45.5%) of ILD seen in our cohort. In a recent study of 146 histological cases in pSS, NSIP comprised 45% of all ILD cases.^18^ However, in a study from Italy comprising of 34 patients, UIP pattern of ILD was more prevalent comprising 38% of the cohort.^19^ The time of onset of ILD is variable, reported differently in different studies. Older series have mainly reported the simultaneous diagnosis of ILD and pSS or development of ILD after the pSS diagnosis.^15^,^20^ Interestingly in our study, we found that ILD preceded pSS diagnosis in 2 patients. A similar observation was seen in a series by Roca et al.,^21^ in which 25% of patients (4 patients) had ILD preceding pSS diagnosis. The majority of patients in our series were symptomatic for ILD (84.8%), which is similar to other series.^21^

ILD in pSS is a condition that influences prognosis and survival**,** however, there are no evidence-based guidelines for management, with available data being limited to case series and case reports. In a retrospective study conducted by Amlani et al.,^22^ out of 19 patients of pSS with ILD, 7 patients received MMF, 7 received Azathioprine, 6 patients received Rituximab, and 5 patients did not receive any sort of immunosuppressive agent. Patients on MMF had significant improvement in FVC%, whereas, on Azathioprine, there was a trend towards improvement. In another retrospective cohort study by Sogkas et al.^23^, Cyclophosphamide and Azathioprine were most commonly used. All of our patients received steroid-sparing agents, with MMF being the most common agent used. MMF is the preferred agent used in our institution because of our vast experience of safety and efficacy with this drug. Only a few investigators have looked into the outcomes of ILD, where worsening was reported in 7–33 % of patients despite treatment.^24^,^21^ Yazisiz et al. reported that ILD was an independent predictor of mortality and the mortality rates ranged from 7 to 39% over 2 to 8 years.^25^ However, our study showed better outcomes, with deterioration seen in only 3 patients (12%). The aggressive approach in the treatment could have been one of the many reasons for the same. This is one of the largest studies showing the efficacy of MMF in pSS related ILD. Two of our patients had successful outcomes with Rituximab therapy, the efficacy of which has been discussed previously.^26^ Chen et al. reported improvement in clinical symptoms and DLCO in 10 patients at 6 months after receiving Rituximab.^26^ In previous studies, not all patients have received steroid-sparing agents unlike in our cohort (**Table 3**). In a retrospective study of 33 patients, the overall five-year survival rate was 87 percent.^18^ The Norwegian systemic connective tissue disease (CTD) and vasculitis registry (NOSVAR), documented a 4 fold higher risk of death in PSS patients with ILD, when followed up for 10 years.^19^ Interestingly we had only one mortality in our cohort, due to sepsis. All of our patients were given pneumococcal and influenza vaccination before the initiation of therapy and co-trimoxazole prophylaxis which could have led to lesser infection rates.

**Table 3.** Previous studies on primary Sjögren's syndrome related ILD with treatment and outcomes.

**Author**	**No. of patients**	**Immunosuppressive medication**	**N (%) no. of patients with steroid-sparing agents**	**Outcomes**	**Mortality**
Roca et al., ^21^ 2016	21	Azathioprine, Cyclophosphamide, Methotrexate, Rituximab	6 (28%)	7 (36.8%) had deterioration	3 (14.3%), lung complications, one unrelated
Shi et al., ^17^ 2009	14	Steroids, Cyclophosphamide	14 (100%)	No worsening	1 (14%), progression of ILD
Parambil et al., ^24^ 2006	18	Steroids, Azathioprine, Cyclophosphamide	5 (27%)	5 (27%) had worsening	7 (39%), progression of ILD
Amlani et al., ^22^ 2020	19	Steroids, Azathioprine, Mycophenolate, Rituximab	14(73%)	3 (15%) had worsening	4 (21%), sepsis, ILD exacerbation, unknown in 2 patients
Yazisiz et al., ^25^ 2020	47	Steroids, steroid sparing agents^*^	NA	NA	16 (34%)
Manfredi et al., ^19^, 2021	34	Steroids, Mycophenolate, Azathioprine	NA	NA	NA
Our Study, 2023	33	Steroids, Azathioprine, Mycophenolate, Rituximab, Cyclophosphamide	33 (100%)	4 (17%) had worsening	1 (3%) due to sepsis

ILD: Interstitial lung disease; NA: Not available.

* Details not mentioned

Our study had several limitations. It was a retrospective study with a small sample size. Due to the small sample size, a comparison of efficacy between the individual therapeutic agents could not be done. Also, the follow-up period was short and not uniform for all patients. There were missing spirometry and HRCT on follow-up for some patients, for which a response criteria protocol was formulated. Also, we did not have a control group for comparison of outcomes.

## CONCLUSION

Immunosuppression with steroids along with steroid-sparing agents led to good clinical outcomes of ILD in a majority of the patients. Mycophenolate mofetil was the commonest steroid sparing agent used in our cohort. Further controlled trials are needed to compare the different treatment options and duration of treatment.

## OPEN DATA SHARING POLICY

The raw data is available with the authors and will be made available on request if needed.
